# The Effects of Overexpression of Histamine Releasing Factor (HRF) in a Transgenic Mouse Model

**DOI:** 10.1371/journal.pone.0011077

**Published:** 2010-06-11

**Authors:** Yueh-Chiao Yeh, Liping Xie, Jacqueline M. Langdon, Allen C. Myers, Sun-Young Oh, Zhou Zhu, Susan M. MacDonald

**Affiliations:** 1 Department of Medicine, Johns Hopkins University School of Medicine, Baltimore, Maryland, United States of America; 2 Graduate Institute of Natural Healing Sciences, Nanhua University, Chiayi, Taiwan; 3 Aurora Sinai Medical Center, Milwaukee, Wisconsin, United States of America; 4 The Johns Hopkins Asthma and Allergy Center, Baltimore, Maryland, United States of America; Ludwig-Maximilians-Universität München, Germany

## Abstract

**Background:**

Asthma is a disease that affects all ages, races and ethnic groups. Its incidence is increasing both in Westernized countries and underdeveloped countries. It involves inflammation, genetics and environment and therefore, proteins that exacerbate the asthmatic, allergic phenotype are important. Our laboratory purified and cloned a histamine releasing factor (HRF) that was a complete stimulus for histamine and IL-4 secretion from a subpopulation of allergic donors' basophils. Throughout the course of studying HRF, it was uncovered that HRF enhances or primes histamine release and IL-13 production from all anti-IgE antibody stimulated basophils. In order to further delineate the biology of HRF, we generated a mouse model.

**Methodology/Principal Findings:**

We constructed an inducible transgenic mouse model with HRF targeted to lung epithelial cells, via the Clara cells. In antigen naïve mice, overproduction of HRF yielded increases in BAL macrophages and statistical increases in mRNA levels for MCP-1 in the HRF transgenic mice compared to littermate controls. In addition to demonstrating intracellular HRF in the lung epithelial cells, we have also been able to document HRF's presence extracellularly in the BAL fluid of these transgenic mice. Furthermore, in the OVA challenged model, we show that HRF exacerbates the allergic, asthmatic responses. We found statistically significant increases in serum and BAL IgE, IL-4 protein and eosinophils in transgenic mice compared to controls.

**Conclusions/Significance:**

This mouse model demonstrates that HRF expression enhances allergic, asthmatic inflammation and can now be used as a tool to further dissect the biology of HRF.

## Introduction

We identified a histamine releasing activity that was found in late phase fluids from nasal lavages, bronchoalveolar lavage (BAL) fluids and skin blister fluids that directly induced histamine release from basophils isolated from a subpopulation of allergic donors [1]. After purification and cloning, this histamine releasing factor (HRF) was found to be identical to translationally controlled tumor protein (TCTP), which is also known as p23 [2]–[4]. This recombinant molecule was found to have the same properties as the originally described HRF derived from nasal secretions, namely, an ability to induce histamine release from selected donors, HRF-responders (HRF-R). This protein is ubiquitously expressed as an intracellular protein. HRF has no leader sequence, therefore, how it gets secreted was elusive until Amzallage et al documented that HRF or as it is alternatively known, TCTP, was secreted by an ER/Golgi-independent route [5]. Furthermore, they documented that secreted HRF/TCTP comes from a pre-existing intracellular pool and co-distributes with TSAP6, a member of a family of proteins that are involved in vesicular trafficking and secretory processes [6], [7].

Homologs of HRF have been described in parasites including *Plasmodium falciparum*, *Wucheria bancrofti*, *Brugiia malea* and *Schistosoma Mansinai*, all of which possess mast cell/basophil histamine releasing activity [8]–[10]. HRF was initially described as a complete secretogogue for histamine and IL-4 secretion from basophils of allergic donors [11]. Initially, it was thought that these donors had a certain type of IgE that interacted with HRF to induce secretion [2]. However, it was subsequently demonstrated that HRF primed all basophils for histamine release as well as IL-4 and IL-13 secretion regardless of the type of IgE [12]. Additional studies demonstrated that HRF did not appear to directly interact with IgE [13], [14]. HRF was also shown to stimulate eosinophils to produce IL-8 and induce an intracellular calcium response [15]. Moreover, HRF has been shown to inhibit cytokine production from stimulated primary T cells and the Jurkat T cell line [16] at the level of gene transcription [16]. Furthermore, Kang et al have identified this molecule as a B cell growth factor [17]. This group demonstrated that HRF bound to B cells and induced cytokine production from these cells. More recently, HRF was shown to stimulate bronchial epithelial cells to produce IL-8 and GM-CSF [18]. Thus, HRF, in addition to functioning as a histamine releasing factor, can modulate secretion of cytokines from human basophils, eosinophils, T cells, B cells and epithelial cells, firmly establishing HRF's extracellular role.

The importance of the association of HRF with human allergic disease has been previously documented in numerous publications. It should be noted that while these observations were made with crude HRF, the same exists with recombinant material. For instance, HRF has been found in human respiratory secretions (BAL) and skin blister fluids [1]. Furthermore, sensitivity to HRF was restricted to a subpopulation of atopic individuals [19]. In a separate study of ragweed allergic patients, there was a significant correlation between the intensity of symptoms in the late phase reaction and basophil histamine release to HRF [20]. In a third study, only basophils from allergic asthmatics and not non-allergic asthmatics responded to HRF. Of those allergic subjects who responded *in vitro*, methylcholine sensitivity and symptoms of asthma were highly correlated [21]. Sampson et al have shown that production of HRF also is associated with clinical status of food allergy and atopic dermatitis [22]. Based on these observations, we believe that HRF may be an important element of the pathogenesis of asthmatic, allergic diseases. Since HRF is present in late phase reaction fluids *in vivo*, it may be contributing to mediator release that is found in the late response. Further understanding of the biology of HRF may help explain the varying severities of allergic disease.

Although HRF has been extensively investigated for many years, most studies have been carried out in primary human or cultured cells. Currently, there is no established animal model available to further explore the function of HRF. One group from Taiwan generated HRF knockout mice by targeted gene disruption [23]. However, HRF knock out mice were embryonic lethal. Since HRF is ubiquitous and highly conserved, our approach has been to create an inducible HRF mouse model using the Tet-On system. Since we wanted to target HRF to the lungs, we used the CC10 promoter that is expressed in Clara cells of the lung epithelium. Here, we report the phenotype of this HRF-inducible mouse.

## Materials and Methods

### Ethics Statement

All procedures performed on mice were in accordance with the National Institutes of Health guidelines for humane treatment of animals and were approved by the Johns Hopkins University Institutional Animal Care and Use Committee (ACUC). The ACUC protocol number is MO07M197.

### Transgenic TRE-HRF-EGFP plasmid construction

The HRF transgenic plasmid was generated by the combination of three main components. The first component is the pTRE-tight vector, which contains a modified TRE (tet response element) controlling the inducible expression of the gene of interest. The second component is human HRF cDNA, which was cloned from U937 cells by RT-PCR and confirmed by sequencing. The third component is the pIRES2-EGFP vector (Clontech, Mountain View, CA). The IRES2 (internal ribosome entry site) allows the enhanced green fluorescent protein (EGFP) gene to be expressed individually as a reporter protein along with HRF in order to facilitate the recognition of expression of transgenic human HRF.

### Generation of transgenic mice

Transgenic HRF mice were generated by pronuclear injection of the transgenic plasmid described above by the John Hopkins Transgenic Core. HRF transgenic mice were crossbred with CC10-rtTA mice on a C57BL/6 background to obtain double transgenic CC10-rtTA/HRF mice (transgene++) for the functional experiments. All experiments shown in these studies were completed during crossbreeding to reach pure background at generation 10. Therefore each protocol was completed with littermate control mice to account for generational variety. Mice were used at 6–11 weeks of age. All mice were housed in cages with microfilters in a specific pathogen-free environment.

### Identification of transgenic mice

The presence or absence of the transgene in the resulting animals and their progeny was determined using tail DNA and PCR analysis. The primer sets used for PCR were EGFP-F, 5′-GAC GTA AAC GGC CAC AAG TT-3′; EGFP-R, 5′-GAA CTC CAG CAG GAC CAT GT-3′; TRE-HRF-F, 5′-GTG TAC GGT GGG AGG CCT AT-3′; TRE-HRF-R, 5′-GTT TCC TGC AGG TGA TGG TT-3′; and CC10-F, 5′-ACT GCC CAT TGC CCA AAC AC-3′; CC10-R, 5′-AAA ATC TTG CCA GCT TTC CCC-3′. The following PCR protocol was used: 95°C for 5 min; 30 cycles of 95°C for 45 sec, 62°C (EGFP and HRF) or 60°C (CC10) for 45 sec, and 72°C for 45 sec; and a final extension at 72°C for 5 min.

### Experimental design

1. Induction of transgene overexpression-Protocol I
  All TRE-HRF-EGFP transgenic mice and CC10 control mice were maintained on normal water until they were 6 weeks old. Then doxycycline (Dox) water at 1 mg/ml in 4% sucrose kept in dark bottles to prevent light-induced degradation was administrated for 3–4 weeks. Regular drinking water was given to littermate controls for comparison for the duration of the experiment (n = 4−11 per group).
2. Ovalbumin (OVA) or PBS sensitization followed by OVA challenge-Protocol II
  The sensitization and challenge protocol was completed as previously described [24]. Briefly, all mice were administrated Dox (1 mg/ml in 4% sucrose) in water on the first day of the experiment (day -7) and through out the challenge protocol to control for nonspecific Dox effects. Mice were divided into four groups (n = 6−9 per group): transgene++ and CC10 littermate sensitized with either OVA (Sigma, St Louis, MO) or PBS on day 0 by intraperitoneal (i.p.) injection of OVA (20 µg OVA adsorbed to 4 mg aluminum hydroxide) or PBS as a control and then boosted with OVA or PBS at day 5. Seven days later, all mice received a daily intra nasal challenge of OVA (20 µg) for three consecutive days.
3. OVA sensitization followed by OVA (or PBS) challenge-Protocol III
  To further explore the role of HRF in the allergic diathesis we design additional experiments that compared effects of HRF on mice that are sensitized with OVA and then challenged with OVA (as previously described in 2) or with sham challenge of PBS (n = 6−8 per group). The protocol was otherwise the same as previously described.

### Assessment of airway hyperresponsiveness (AHR)

Airway hyperresponsiveness (AHR) was assessed by methylcholine-induced airflow obstruction from conscious unrestrained mice placed in a whole body plethysmograph (model PLY 3211, Buxco Electronics Inc., Troy, New York, USA) [25]. In brief, mice were placed into whole-body plethysmographs and interfaced with computers using differential pressure transducers. Mice were challenged for 3 min with a series of aerosolized methylcholine inhalations. Enhanced pause (Penh) was monitored for 3 min after each aerosol challenge by transducer (model TRD 5100, Buxco Electronics Inc., Wilmington, NC) connected to preamplifier modules (model MAX2270, Buxco Electronics Inc., Willington, NC). Penh is a function of total pulmonary airway during the respiratory cycle and is described by the following equation: Penh = pause x (PEP/PIP), Pause, PEP and PIP are expiration time, the peak expiratory pressure, and peak respiratory pressure, respectively.

### Lung and bronchoalveolar lavage samples

Lung tissues and BAL samples were obtained as previously described [26]. Briefly, mice were anesthetized, the trachea was isolated by means of blunt dissection, and small-caliber tubing was inserted and secured in the airway. Two successive volumes of 1 ml of PBS were instilled and gently aspirated and pooled. The BAL fluids were immediately centrifuged at 6000 rpm at 4°C for 5 min, and supernatants were stored at −80°C until use. After removing the supernatant, the cells were counted manually in an Improved Neubauer (Hausser Scientific, Horsham, PA) chamber. Cytocentrifuged preparations (Cytospin 2, Cytospin, Shandon, UK) were stained with May-Grünwald-Giemsa for differential cell counts and examined under bright-field optical microscopy using a light microscope, and corresponding digital images were captured for subsequent analysis by a Spot CCD Camera driven by Advanced Spot RT Software version 3.3 (Diagnostic Instruments Inc., MI, USA). The lung was perfused with PBS through the right ventricle until the lung was clean. The lung was excised for RNA and protein analyses and/or inflated with fixative for histology.

### Western blot analysis

Lung tissues were lysed with a lysis buffer (62.5 mM Tris-HCl pH 7.5; 500 mM NaF; 100 mM Na_3_VO_4_, and proteinase inhibitor cocktail, BD BioSciences, San Jose, CA) and then centrifugated at 10,000×g at 4°C for 30 min to obtain the cellular proteins in the supernatant. The protein concentrations were determined by BCA Protein Assay (Pierce Biotechnology Inc., Rockford, IL), and 20 µg of total protein from each sample were boiled for 5 min and electrophoresed on 4–20% Tris-Glycine gels (Invitrogen, Carlsbad, CA), transferred to nitrocellulose membranes, and blocked in blocking buffer (150 mM NaCl in 10 mM Tris, pH 7.5 containing 5% non-fat dry milk) for 1 hr at room temperature. The membranes were blotted with anti-EGFP (1∶200; Santa Cruz Biotechnology, CA) or anti-HRF (in house prepared monoclonal antibody [27]) at 4°C for at least 16 hours, washed three times (20 mM Tris-HCl, pH 7.5, 137 mM NaCl, and 0.1% Tween 20), incubated with HRP-conjugated goat anti-mouse IgG secondary antibody (1∶5000 dilution, GE Healthcare,UK) for 1 h at room temperature, washed three times, followed by the detection of signal with SuperSignal® West Pico Chemiluminescent Substrate (Pierce Chemical Co., Rockford, IL, USA). The density of each protein band was scanned using the Bio-Rad Gel Doc system and the Quantity One 4.4.1 software (Bio-Rad Laboratories, Hercules, CA) and compared by densitometry to positive control.

### Immunofluorescence microscopy

Freshly dissected lungs were fixed in Protocol Safefix II (Fisher Scientific Co., Kalamazoo, MI) and embedded in paraffin. The lung tissue sections (5 µm in thickness) were deparaffinized with xyline, rehydrated gradually with graded alcohol solution (100%, 95%, and 80%), and then washed with deionized water and immersed in 3% BSA for 1 h to block nonspecific binding. These slides were then incubated with primary mouse anti-HRF antibody at dilutions of 1∶200 for 18 h at 4°C, washed twice in PBS/Tween-20 solution, incubated with a Texas Red-conjugated secondary antibody for 1 h at room temperature, and photographed with a fluorescent microscope. We chose anti-HRF antibody to directly measure HRF. Therefore, an anti-GFP antibody was unnecessary.

### Histological examinations of lung tissues

Lung tissues were fixed in Protocol Safefix II (Fisher Scientific Co.), and processed by AML Laboratories (Baltimore, MD). Briefly, samples were embedded with paraffin, sectioned at 5 µm, deparaffinized, dehydrated, and stained with hematoxylin and eosin (H&E). In collaboration with Dr. Allen Myers, Head of the Johns Hopkins Histologic Core, these specimens were examined under bright-field optical microscopy using a light microscope, and corresponding digital images were captured for subsequent analysis by a Spot CCD Camera driven by Advanced Spot RT Software version 3.3 (Diagnostic Instruments Inc.).

### Reverse transcription polymerase chain reaction (RT-PCR)

Lungs were rapidly dissected and frozen on dry ice. For extraction of total RNA, tissues were homogenized in 1 ml of ice-cold TRIzol reagent (Invitrogen) and the total RNA was reverse-transcribed into cDNA using a random hexamer and a GeneAmp RNA PCR Kit (Applied Biosystems, Foster City, CA). PCR analysis was performed by AccuPower PCR PreMix kit (BIONEER, Alameda, CA) with each amplified primer set (EGFP-F, 5′-GAC GTA AAC GGC CAC AAG TT-3′; EGFP-R, 5′-GAA CTC CAG CAG GAC CAT GT-3′; actin-F, TCC TGT GGC ATC CAG GAA ACT; actin-R, GGA GGA ATG ATC CTG ATC TTC; IL-4-F, 5′-TCA TCG GCA TTT TGA ACG AG-3′; IL-4-R, 5′-GAA TCC AGG CAT CGA AAA GC-3′; IL-13-F, 5′-TCA GCC ATG AAA TAA CTT ATT GTT TTG T-3′; IL-13-R, 5′-CCT TGA GTG TAA CAG GCC CAT TCT-3′; MCP-1-F, 5′-ACC AGC CAA CTC TCA CTG AAG C-3′; MCP-1-R, 5′-CAG AAT TGC TTG AGG TGG TTG TG-3′; MDC-F, 5′-CCT GGT GGC TCT CGT CCT TC-3′; MDC-R, 5′-CAG GGG ATG GAG GTG AGT AA-3′) under formulated conditions. Amplified PCR products were analyzed by means of electrophoresis, and the intensity of the bands and the ratio of specific mRNA to β-actin were analyzed with the Bio-Rad Gel Doc system and the Quantity 4.4.1 software (Bio-Rad Laboratories).

### Quantification of total IgE, OVA-specific IgE, and chemokine levels

Blood drawn from the heart and BAL fluids were used for measurement of total IgE levels with commercial mouse IgE isotype-specific enzyme-linked immunosorbent assay (ELISA) according to the manufacturer's instructions (BD Biosciences, San Diego, CA). The levels of OVA-specific IgE in serum were determined using a commercial ELISA per the manufacturer's instructions (MD Biosciences, Zürich Switzerland). IL-4 and eotaxin levels of BAL fluids were quantitated using commercial ELISA kits (R&D System, Inc, Minneapolis, MN) according to the manufacturer's instructions.

### Statistical analysis

All experiments were repeated with multiple mice and matched littermate controls as indicated by the n values in each experiment. The quantitative data of continuous variables were expressed as mean ± S.E.M of each group. The statistical difference between experimental groups was determined by Student's *t* test. A *p* value of less than 0.05 was considered statistically significant.

## Results

### Over Expression of Transgene and Phenotypic Analysis (Protocol I)

#### Transgene expression

**Figure 1:**
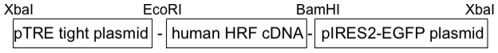
Schematic of the Physical Map of the HRF Transgene. The plasmid construction is described in the Materials and Methods. TRE (tet response element), pIRES (internal ribosome entry site), EGFP (enhanced green fluorescent protein).

The schematic of the physical map of the transgene is shown in Figure 1. We specifically did not analyze copy numbers and integration sites of the transgene. Using a combination of endonuclease digestion, PCR and nucleotide sequencing, transgene integration sites in the genome can be determined. However, knowing where the transgene is does not prevent its segregation from its surrounding genes in the offspring, and there are no practical values in the function of the transgene. Ultimately the expression and function of the transgene needs to be tested experimentally. The same is true with the copy numbers of the transgene. Additionally, correlation between copy numbers and expression and function of a transgene is not very clear.

**Figure 2:**
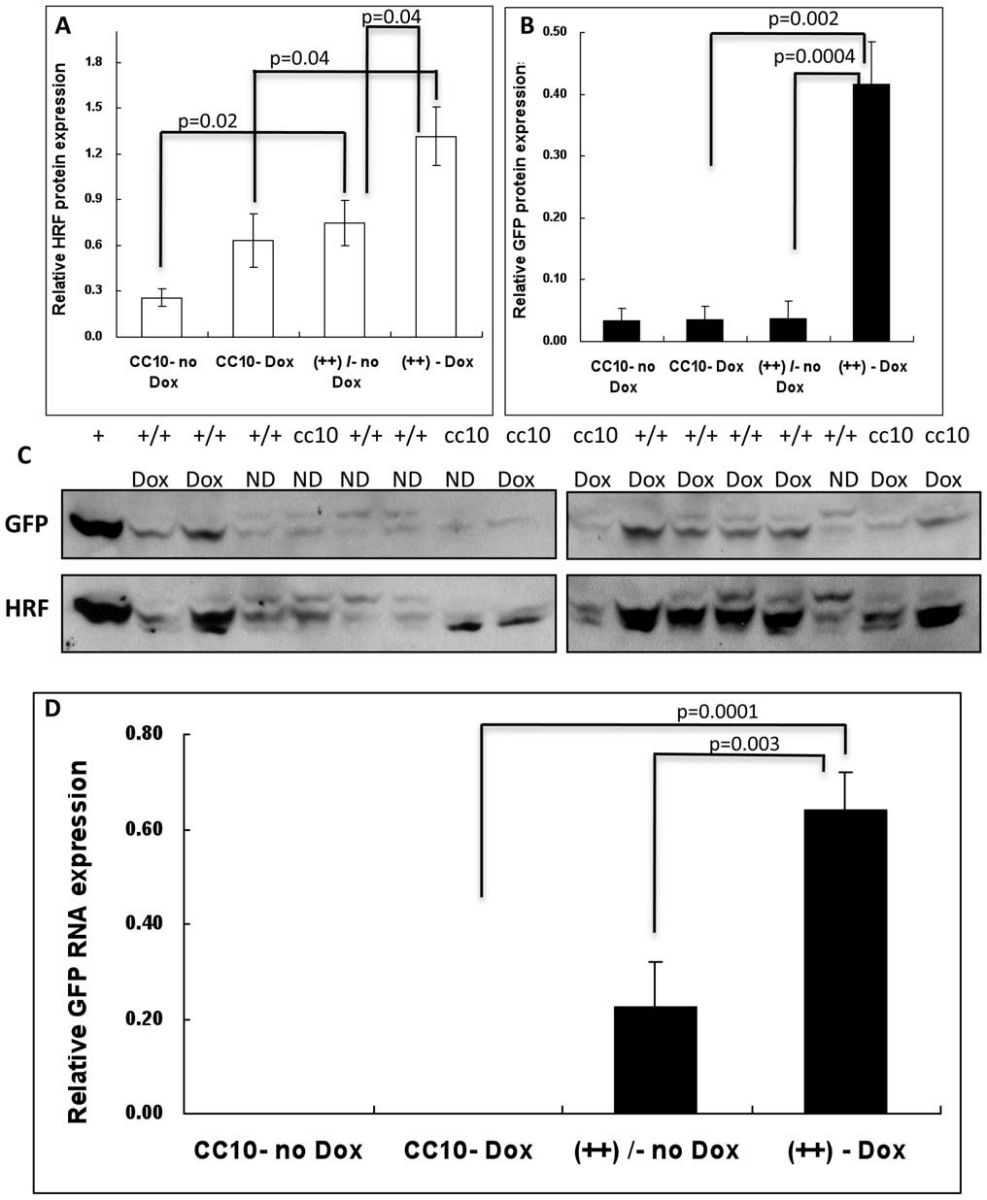
Transgenic Protein and mRNA Expression for Mice in Protocol I. In all cases CC10 are control mice either fed Dox (Dox) or fed regular water (no Dox). Transgenic mice (++) are either fed Dox (Dox) or are on regular water (no Dox). Each group has between 4 and 11 mice (backcrosses 5–8). Panel A depicts the relative amount of HRF protein based on densitometric analysis of Western blots. Panel B is the relative amount of GFP protein based on Western blots. Relative protein levels are expressed as a function of the positive control of each protein. Panel C is a representative Western blot in the transgenic mice (++) or littermate controls (CC10) in the presence of doxycycline (Dox) or absence of doxycycline (ND). Panel D shows mRNA for GFP relative to the housekeeping gene, beta actin.

After crossbreeding, both transgene++ and CC10 control mice were treated with Dox at 1 mg/ml for 3–4 weeks. Regular drinking water was also given to littermate controls for comparison. Transgene expression was verified by both Western blot and mRNA analysis of lung tissue. First, lung tissue lysates were analyzed by Western blot for both EGFP and HRF expression, as shown in Figure 2. The homology between mouse and human HRF yields a cross-reaction and therefore Western blots could not distinguish between native and transgenic HRF expression, therefore, GFP was used as an additional marker of transgene expression. Expression of HRF (Figure 2, Panel A) as well as over expression of GFP (Figure 2, Panel B) in Dox treated mice indicated that the Tet on system was operating effectively. However we did see a modest, but not statistically significant nonspecific Dox effect on HRF expression in CC10 mice. Furthermore, there was a Dox-independent induction of HRF when CC10 and ++ mice were fed with normal water (p = 0.02). However, GFP expression (Panels B and D) was not significantly increased among these groups. Therefore, we do not think there is evidence of promoter leakiness. Nevertheless, subsequent studies were done comparing transgene++ and CC10 littermate controls all fed Dox. RT-PCR analysis of lung tissues for EGFP expression confirmed these results (Figure 2, Panel D). Of note, a representative Western blot in the transgene++ or CC10 littermate controls in the presence or absence of doxycycline is shown in Figure 2, Panel C.

#### Phenotypic analysis

**Figure 3:**
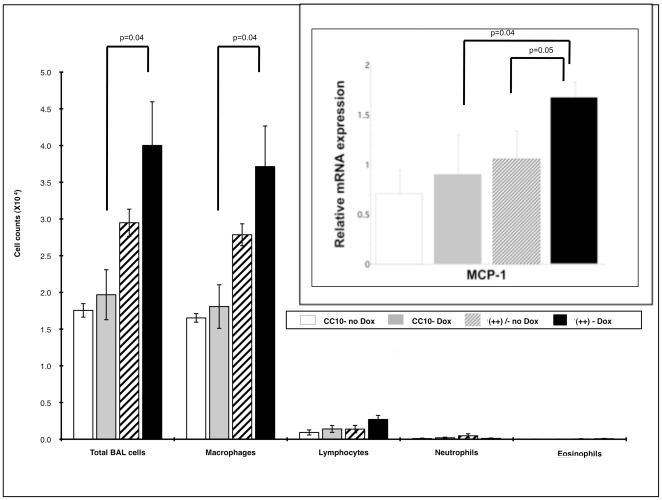
BAL Cell Counts for Mice in Protocol I. The average cell counts +/− SEM from BAL supernatants for each group of mice is shown. Total cells were counted using Erythrosin B stained under light microscopy. Cell differentials were done using cytospin slides of the BAL stained with Dif-Quik. The insert shows mRNA for MCP-1 between various groups of mice. In all cases each group had between 4 and 11 mice (backcrosses 5–8).

BAL cells were examined and there was a significant increase in total cell counts in transgene++ mice compared to CC10 mice following dox induction and the change was primarily due to an increase in macrophages (p = 0.04, Figure 3). Of note, there are significant differences (p<0.001) between transgenic ++ mice and CC10 littermate controls not fed Dox in both total cells and macrophages. This may be due to the increased HRF expression noted in Figure 2. However, given that the Dox induction between transgenic ++ and control CC10 mice was significantly different, we proceeded with experiments in which all mice were fed Dox, as previously noted. Due to the increase in macrophages, we investigated mRNA levels for MCP-1. MCP-1 is known to be involved in the initiation of inflammation [28]. As shown in the insert of Figure 3, there was a significant increase in mRNA levels for MCP-1 in transgenic++ mice fed Dox as compared to CC10 mice fed dox (p = 0.04) and to transgenic++ mice fed regular water (p = 0.05). Markers of TH2 inflammation were also examined, including serum and BAL IgE levels, eotaxin, IL4, IL5 and IL13 expression in lung tissues, but there were no differences seen (data not shown). This is not surprising based on the lack of TH2 cellular infiltrate in the lungs of the transgene++ mice (Figure 3), and the previously published studies. Specifically, Teshima et al found that HRF caused eosinophil recruitment in sensitized but not normal mice [29]. Additionally, Kang *et al* found that *in vivo* administration of HRF increased total and Ag-specific Ig synthesis, but these studies were done in the more conventionally allergic BALB/c mice [17]. Thus, we had a modest phenotype when the HRF transgene was turned on, but it was not the allergic phenotype we initially expected. Therefore, we proceeded to use our transgene++ mice in OVA sensitization and challenge experiments to see if HRF enhanced the allergic phenotype.

### OVA (or PBS) Sensitization followed by OVA Challenge (Protocol II)

The sensitization and challenge protocol was essentially the same as described by Brusselle et al [24] and is outlined in Material and Methods section. All mice were fed Dox 7 days prior to and throughout the challenge protocol to control for nonspecific Dox effects. The mice were divided into 4 groups: transgene++ and CC10 littermates sensitized with either OVA or PBS as a control (n = 6−9 per group). This model has been used extensively and has generated brisk and consistent TH2 responses in the murine lung [26]. After intranasal challenge, the phenotype of each mouse was also assessed by BAL cell counts, histopathology of bronchi and trachea, serum and BAL IgE levels, allergic cytokine gene and protein expression in the BAL and/or lung tissue.

#### Transgene expression

**Figure 4:**
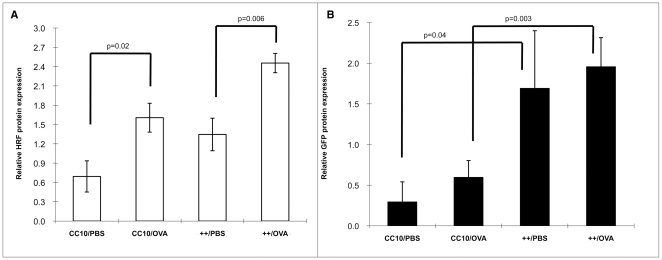
Transgenic Protein Expression for Mice in Protocol II. Panel A depicts the relative amount of HRF protein based on densitometric analysis of Western blots. Panel B is the relative amount of GFP protein based on Western blots. Relative protein levels are expressed as a function of the positive control of each protein. In all cases mice were sensitized with PBS or OVA, then all mice were challenged with OVA. Each group had between 6 and 9 mice (backcrosses 3–6).

Transgene expression was confirmed in each mouse as previously described and is shown in Figure 4. Of note, HRF protein levels (Panel A) were increased following OVA sensitization and challenge compared to PBS sensitized OVA challenged litter mate controls in transgene++ mice (p = 0.006) and in CC10 controls (p = 0.02). The increases in HRF are endogenous, and not promoter driven transgenic HRF based on the similar GFP levels in the groups (Figure 4, Panel B). This indicates that OVA sensitization increases naturally occurring HRF, and supports HRF's role in the antigen driven allergic phenotype.

**Figure 5:**
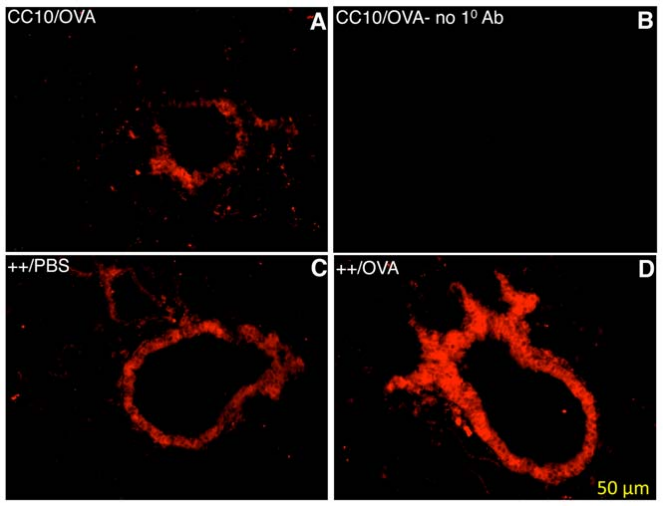
Immunoflourescent Staining for HRF in Mouse Lung. The bronchi of 3 mice fed Dox to induce over expression of HRF are shown. The genotype and sensitization reagent for each mouse is shown in the top left corner of each panel. Panels A, C and D are stained with rabbit anti-HRF antibody. Panel B illustrates the nonspecific staining with secondary antibody alone.

Lung cross-sections were subjected to immunoflourescent staining to visualize HRF expression localized to the lung epithelium. Immunoflourescent staining shown in Figure 5, illustrates that HRF is up-regulated in the epithelium of transgene++ mice fed Dox (Panels C and D) compared to littermate controls (Panel A). Furthermore, OVA sensitization up-regulates HRF in the lung epithelium (Panel D, compared to Panel C). Nonspecific staining with no primary antibody (Panel B), as well as additional experiments with an irrelevant antibody (not shown) demonstrated no non-specific staining. The increase in HRF expression could be due to an increase in the number of Clara cells following antigen challenge or an increase in the capacity of each cell. The images in Figure 5 indicate that either could be true.

**Figure 6:**
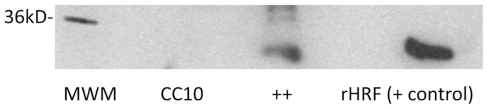
Expression of HRF Protein in BAL. Transgenic HRF mice (++) and littermate control (CC10) mice were treated with doxycycline water, sensitized and challenged with OVA, as previously described. BAL supernatants were collected and pooled with 9 mice per pool, the IgG was precleared by incubation with Protein A beads, and then the supernatants were concentrated with a Centricon to 50-fold above the original pool/volume. Concentrated samples were run a tris-glycine gel with a recombinant HRF positive control. Western blotting was completed using a monoclonal anti-HRF, as described for the whole lung lysates.

HRF is an abundant cellular protein and has been implicated in cell cycle-related activities [30], but there are numerous reports of its extracellular function [2], [8]–[10], [15]–[18]. In order to demonstrate that HRF was additionally secreted in our mouse system, we concentrated BAL samples 50-fold from OVA challenged transgenic mice and compared to concentrated BAL from OVA challenged CC10 littermate controls. The results of the Western blot are shown in Figure 6. HRF expression levels in the transgenic mice is greater than that from CC10 littermate controls. While HRF is not visible in the controls due to the level of detection, these expression levels of HRF in all BAL from transgenic mice are consistent with the literature. In that case, a 100-fold concentration was necessary to detect HRF in human BAL of asthmatic patients [18]. This demonstrates that HRF is secreted and available to act extracellular to activate cells. Thus, our transgenic mouse provides both an intracellular and extracellular expression of HRF.

#### Lung Histology

**Figure 7:**
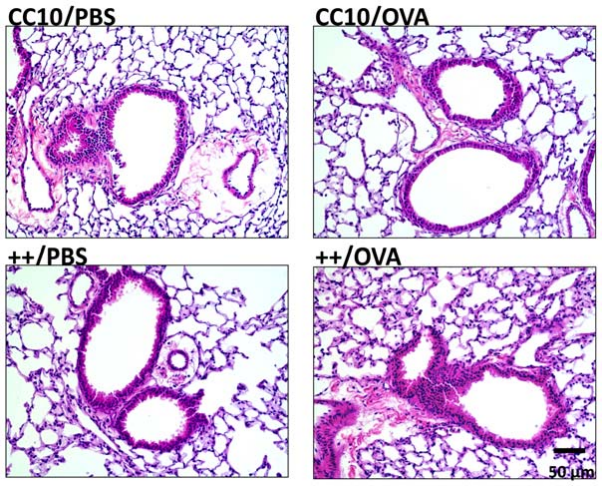
Mouse Lung Histology after OVA Challenge. The mouse lungs were fixed, sectioned and stained with H and E. The genotype and sensitization reagent of each mouse is shown above the corresponding panel.

OVA sensitized and challenged mice demonstrated a marked cellular infiltrate in the bronchus when stained with hematoxilin and eosin, as shown in Figure 7. Eosinophils and enlarged and activated macrophages were seen in transgenic++ mice sensitized and challenged with OVA (++/OVA). This pattern was observed in comparison to CC10 littermate controls treated the same way (CC10/OVA), as well as transgenic++ mice sensitized with PBS (++/PBS). The differences in histology between these groups are concordant with the differences in BAL cell counts.

#### BAL cell assessments

**Figure 8:**
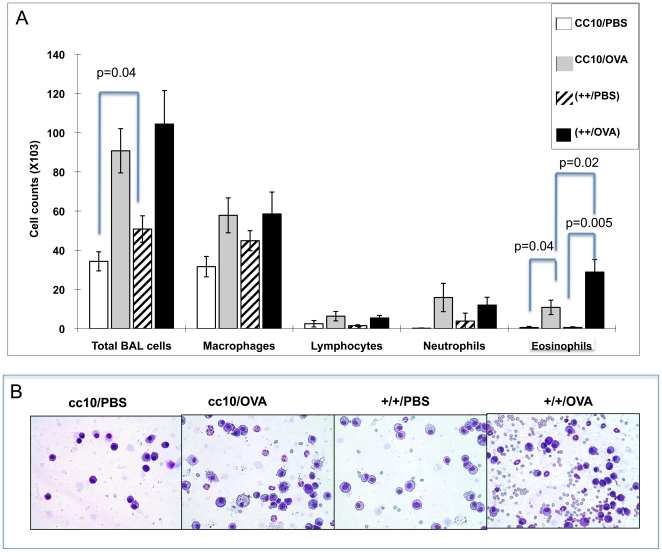
BAL Cell Counts. Panel A depicts the average cell counts +/− SEM from BAL supernatants for each group of mice. Total cells were counted using Erythrosin B stained under light microscopy. Cell differentials were done using cytospin slides of the BAL stained with Dif-Quik. Panel B shows representative cytospins from these mice with genotype and sensitization reagent on the top of the cytospin. N = 6−9 mice per group (backcrosses 3–6).

BAL cells from transgene++ mice showed significant differences in the number of total cells as well as the number of eosinophils that infiltrated the lung (Figure 8 Panel A). Specifically, transgene ++ mice sensitized with PBS(++/PBS), and then challenged with OVA have more total cells than CC10 littermate controls treated the same way(CC10/PBS) (p = 0.04). No significant differences in specific cell types were seen between these 2 groups, although macrophages appear to be primarily responsible, as was found in antigen naïve mice (Figure 3, Panel A). These data recapitulate the previous data from Protocol I indicating that HRF is capable of inducing the start of inflammation by recruiting cells to the lung. These data suggest that HRF operates independently of the effects of sensitization, and perhaps initiates the early steps of inflammation through macrophage recruitment. As expected, mice sensitized and challenged with OVA had increased eosinophils compared to littermate controls that received only PBS sensitization (p = 0.04) and the same was true for the transgene++ group comparisons (p = 0.005). Most importantly, the OVA sensitized and challenged transgenic++ mice (++/OVA) had a significantly larger eosinophil infiltration when compared to the CC10 litter mate controls treated the same way(CC10/OVA) (p = 0.02). These data support the previously published data that HRF is involved with the recruitment of eosinophils to the lung of allergen sensitized mice [29]. Figure 8 Panel B, pictures BAL cells from individual mice and illustrates the pattern seen for the groups as a whole shown in the graph in Figure 8 Panel A.

#### IgE levels

**Figure 9:**
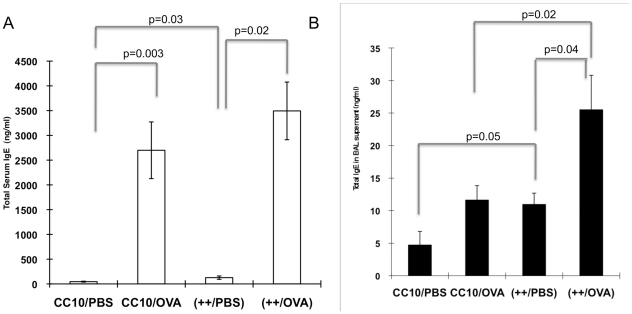
Serum and BAL IgE Levels. Panel A illustrates the average total serum IgE +/− SEM for each group of mice. Panel B is the average IgE levels detected in BAL supernatants from the same mice. IgE levels were measured by ELISA. N = 6−9 mice per group (backcrosses 3–6).

Elevated IgE levels in the serum is a characteristic feature commonly seen in allergic asthma patients and in mouse models of allergen-induced asthma. IgE is important for the functions of cells bearing the high affinity IgE receptor, such as mast cells and basophils. As expected, we found significantly increased serum IgE levels following OVA sensitization and challenge in the transgene++ group and the CC10 group compared to littermate controls sensitized with PBS (p = 0.02 and p = 0.003 respectively, Figure 9, Panel A). Interestingly, transgene++ mice sham sensitized with PBS had significantly higher serum IgE levels than CC10 controls (p = 0.03). Similar to the cell count assessment (Figure 8, Panel A) HRF exerted an effect in the absence of allergen sensitization. There was no significant difference between CC10 and transgene ++ mice challenged and sensitized with OVA, perhaps due to the robust levels induced by the sensitization and challenge. OVA specific IgE was significantly increased following OVA sensitization and challenge, but not changed by HRF expression in the transgene++ mice (data not shown). These data suggest that HRF exerts its effect independently of sensitization and suggest a role in the response phase.

Furthermore, BAL IgE levels were significantly elevated in transgene++ mice compared to CC10 littermate controls in both OVA (p = 0.02) and PBS (p = 0.05) sensitized groups (Figure 9, Panel B). While OVA sensitization and challenge elevated the BAL IgE as expected, HRF over expression in the lung appears to have a greater effect on these levels. Of note, all mice received antigen to the lung and the combination of HRF and antigen gave a strong local effect on BAL IgE. These data are consistant with HRF being important in the response phase and support HRF's role in the allergic inflammation in the lung, given that native HRF is seen in increased levels in the lung following OVA sensitization and challenge (Figure 4).

#### Cytokine levels

**Figure 10:**
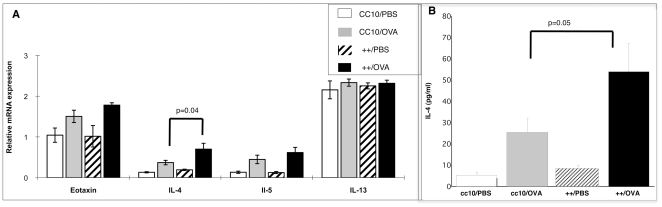
Cytokine and Chemokine Expression Levels. Panel A depicts relative mRNA levels compared to the housekeeping gene, beta actin, using primers specific to each cytokine or chemokine noted. Equal amounts of RNA were used for this relative quantitation. Panel B shows protein levels in BAL supernatants for IL-4 measured by ELISA. N = 6−9 mice per group. Groups of mice are the same as described in Figure Legend 4 (backcrosses 3–6).

Asthma is a chronic inflammatory disorder of the lung and TH2 inflammation of the airway is a major component of asthma. It has been demonstrated in animal models of asthma that allergens can elicit TH2 inflammation and that IL-4 is essential in this response. For example, in the presence of IL-4, naïve CD4+ T cells differentiate into TH2 cells [31]. In order to investigate the underlying molecular mechanism of the augmented pulmonary inflammation in the HRF-inducible transgenic mouse we investigated several cytokine and chemokine expression profiles in the lung. As shown in Figure 10, when mice were sensitized and challenged with OVA, transgene++ (++/OVA) mice had greater mRNA levels of IL-4 (p = 0.04) than CC10 littermate controls treated the same way (CC10/OVA). There were no differences observed in mRNA levels for eotaxin, IL-5 or IL-13 between these groups (Figure 10). Protein levels for IL-4 were also measured from BAL supernatants (Figure 10 Panel B). IL-4 protein levels were consistent with the mRNA levels and were significantly increased in both CC10 and transgene++ mice following OVA sensitization and challenge. Moreover, following OVA sensitization and challenge transgenic mice (++/OVA) had significantly more IL-4 protein than CC10 mice (CC10/OVA) (p = 0.05). The up regulation of IL-4 as evidenced by both mRNA and protein in the presence of HRF expression illustrates HRF's role in stimulating other cytokines central to the disease process.

#### Airway hyperreactivity (AHR)

Airway physiology abnormalities are a hallmark of asthma in human and animal models of asthma. Methylcholine challenge was used to measure airway reactivity. We saw an increase in airway reactivity following sensitization and challenge with OVA, however, there were no significant differences between littermate controls, except at the top dose (50 mg/ml) between CC10/OVA vs. CC10/PBS sensitized (data not shown). The over expression of HRF either by Dox induced over expression, or by natural allergen induced up-regulation did not appear to significantly effect the airway reactivity to methylcholine compared to that seen with control CC10 mice. This result is not unexpected since it is known that AHR is IL-13 dependent [26] and IL-13 was not elevated in this transgenic mouse model (Figure 10). Furthermore, IL-4, which we do see increased in this model, does not induce AHR to methylcholine. [32]. Since we did not see any changes in AHR using Penh, we choose not to pursue invasive measurements of AHR.

### OVA Sensitization followed by OVA (or PBS) Challenge (Protocol III)

To further explore the role of HRF in the allergic diathesis we designed additional experiments that compare effects of HRF on mice that are sensitized with OVA and then challenged with OVA (as previously done in Protocol II) or with sham challenge of PBS (n = 6−8 per group). The absence of OVA challenge would further dissect the role of HRF in the sensitization and response phases. The protocol and phenotypic analysis were otherwise the same as previously described.

#### Transgene expression

Transgene expression was confirmed in each mouse as previously described and HRF protein was up regulated following Dox administration on transgene++ mice compared to CC10 littermate controls (p = 0.03). Of note again, is that HRF protein levels are increased following OVA sensitization and challenge compared to OVA sensitized PBS challenged litter mate controls in both transgene++ (p = 0.03) and CC10 mice (p = 0.02, data not shown). This indicates that OVA challenge increases naturally occurring HRF, not unlike the results in Protocol II (Figure 4, Panel A) where OVA sensitization increase HRF protein levels. GFP protein levels were not significantly different following OVA challenge (data not shown). This was previously shown in Protocol II and indicates that endogenous HRF, not promoter driven transgenic HRF, accounts for the increase in HRF protein.

#### Phenotype Analysis

Overall, results seen in the Protocol III were similar to the data shown in the previous Protocol II between transgenic++ and CC10 control mice OVA sensitized and challenged. Namely, an increase in airway reactivity to methylcholine challenge following sensitization and challenge with OVA was seen, however, there were no significant difference between littermate controls between transgene++ OVA versus PBS challenge. Also, IL-4 levels and eosinophil numbers were elevated (data not shown). However, total cells and macropahges were not significantly increased as seen in both Protocol I, which are antigen naïve mice, and Protocol II which were not sensitized with antigen. These data indicate that HRF does not affect macrophage recruitment during OVA sensitization, but HRF may initiate inflammation via macrophage recruitment in the absence of sensitization.

#### IgE levels

**Figure 11:**
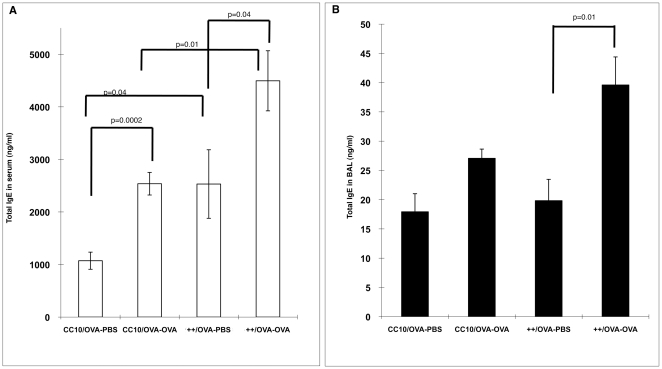
Serum and BAL IgE Levels-Protocol III. Panel A illustrates the average total serum IgE +/− SEM for each group of mice. Panel B is the average IgE levels detected in BAL supernatants from the same mice. IgE levels were measured by ELISA. N = 5−8 mice per group (backcrosses 6–8).

As expected, serum IgE levels significantly increased following OVA sensitization and challenge in both the CC10 controls and the transgene++ group when compared to littermate controls challenged with PBS (Figure 11, Panel A). Transgene++ mice sham challenged with PBS (++/OVA-PBS) had greater serum IgE levels than CC10 controls (CC10/OVA-PBS)[p = .04]. Based on these data, as well as serum IgE levels from Protocol II (Figure 9), HRF can increase serum IgE levels in conjunction with either OVA sensitization or challenge. Interestingly, transgene++ mice sensitized and challenged with OVA (++/OVA-OVA) also had higher IgE levels than CC10 mice treated the same way (CC10/OVA-OVA)[p = .01]. Unlike the data in Protocol II (Figure 9), this difference could be due to increase in strain purity between the protocols i.e. increased strain purity revealed a previously unappreciated significance. Additionally, OVA challenge is necessary for HRF to exert an effect in the response phase as seen in the BAL levels (Figure 11, Panel B). Specifically, there was no increase in BAL IgE in the sham-sensitized transgene++ mice compared to the CC10 mice treated the same way. This is in contrast to the results in Protocol II, where sham-sensitized and OVA-challenged mice showed a significant increase in BAL/IgE in the presence of HRF (Figure 9, Panel B). BAL IgE levels between transgene++ and CC10 mice sensitized and challenged with OVA had the same trend as seen in Protocol II, but did not reach statistical significance (p = .07).

## Discussion

In our transgenic HRF mouse model, we observed an induction of HRF in the lung and corresponding increased numbers of macrophages as well as levels of MCP-1 levels in the lungs. These data were shown in Protocol I (Figures 2 and 3). MCP-1 expression is associated with many inflammatory conditions and notably is elevated in the bronchial epithelium of asthmatics [28]. MCP-1 also is known to be involved with the initiation of inflammation and is a chemoattractant for monocytes and macrophages to areas of inflammation. Additionally, cultured macrophages have been shown to secrete HRF in response to M-CSF [29]. Therefore, activated macrophages could be a source of endogenous HRF found in the lung. In addition, OVA immunized mice challenged with M-CSF caused macrophage infiltration and HRF over production that was similar to OVA challenge. [29]. Furthermore, HRF has been shown to stimulate the secretion of GM-CSF and IL-8 from bronchial epithelial cells [18]. Taken together our data and the previously mentioned studies suggest that HRF may increase airway inflammation by activating cells such as epithelial cells or basophils (see below) to increase cytokine and chemokine production which in turn up regulate the HRF secretion and significantly contribute to the local inflammation in the allergic, asthmatic lung.

We found that HRF levels in the lungs were increased by both OVA sensitization and challenge. This increase was observed in both HRF transgenic mice and CC10 control mice following either OVA sensitization or OVA challenge (see Figure 4). The immunoflourescent staining of the mouse lung (Figure 5) illustrates both an increase in density and intensity of staining suggesting both an increase in the number of cells expressing HRF and the amount of HRF expressed. The increase in expression in transgenic mice could be due to the increase in the number of Clara cells following OVA challenge and sensitization. However, GFP expression levels, as analyzed at protein and mRNA levels, were not increased under these same conditions. Moreover, the increases following OVA sensitization or challenge in the CC10 littermate controls could be due only to endogenous HRF. We hypothesize based on this data as well as the previously mentioned work, that antigen challenge could increase levels of HRF which then acts on other cells to produce inflammatory cytokines.

We also showed after OVA sensitization and challenge that HRF transgenic mice had increased levels of the TH2 cytokine IL4, but not eotaxin, IL-5 or IL-13. IL-4 is necessary for the induction of IgE production and the increases may be responsible for the observed increase in total IgE. In addition, IL-4 has been shown to play an important role in the migration of eosinophils from the lung into the airway [27], [28]. Moreover, previous studies using human cells have shown that HRF is capable of inducing IL-4 from human basophils [8]. In fact, IL-4 as well as IL-13 production and histamine release was observed in response to HRF from a subset of allergic donors basophils [9]. In addition, HRF primed all donors' basophils for IgE dependent mediator release following antigen or anti-IgE stimulation. Not surprisingly, in human work done *in vitro*, HRF has also been shown to cause chemotaxis of eosinophils and secretion of IL-8 from allergic donors [12]. In the present study, the allergen sensitized and challenged mice also had increased numbers of infiltrating eosinophils in their lungs (Figure 8). Although our previous work in human cells showed HRF caused IL-13 secretion, we did not see any changes in mRNA for IL-13 in the lung of transgenic HRF mice, in the presence or absence of OVA sensitization and challenge. IL-13 has been linked to airway hyper responsiveness and mucous production in the same mouse model of allergy [23]. Therefore, given the lack of IL-13 expression in our transgenic mice it is not surprising that we do not see increased AHR or mucous production.

HRF has been shown to be present in the BAL of asthmatic patients [18] as well as in the nasal lavage [1] and skin blister fluids of patients with late phase allergic inflammation [33]. In the present study, we could detect HRF in the BAL of the mice (see Figure 6). The exact cell type that HRF activates in this model is not known. However, given the fact that we see increased IL-4 levels and eosinopils recruited into the BAL, the mouse basophil is a likely candidate. Mouse basophils have been shown to be the cells responsible for secretion of IL-4 [34], [35]. In fact, human and mice basophils have been demonstrated to have more IL-4 per cell than T cells and to be responsible for IL-4 secretion in allergic reactions [36], [37]. Future experiments are designed to test the mechanism of action of HRF in our transgenic mouse model, and we are currently focused on the basophil.

HRF has been previously shown to activate epithelial cells [18] and in our transgenic mouse model, HRF is expressed in the Clara cells of the epithelium. However, Clara cells do not produce IL-4, therefore, the observed increases in IL-4 are most likely due to secreted HRF. Also, as previously mentioned, HRF is known to be secreted in an ER/Golgi-independent manner [5]. Intracellular HRF levels have been associated with increases in tumors [38]. Elevations of intracellular HRF/TCTP that are associated with tumors would not likely occur in the three week time frame that we use in our model, and no gross changes in lung anatomy were observed.

HRF transgenic mice have increased total serum IgE levels following either OVA sensitization or challenge (Figures 9 and 11), suggesting a systemic effect of the over expression of HRF in the lung. We also found increases in BAL IgE in transgenic HRF mice following antigen challenge (Protocol II) in the absence of sensitization, but not in the absence of antigen challenge (Protocol III), suggesting that HRF is active in the response phase. This is in accordance with the observation that OVA specific IgE in the serum is not increased in the HRF transgenic mice following OVA sensitization and challenge when compared to CC10 littermate controls treated the same way. Both observations suggest that HRF elicits its effects in the response and not the sensitization phase.

In summary, endogenous HRF is up-regulated after either allergen sensitization or challenge. Following OVA sensitization and OVA challenge, we see the same trends, namely, increases in IL-4 and eosinophils and no additional increases in AHR. However, there are no differences in total cells and increases in macropages following OVA sensitization and challenge with PBS. This points to the fact that HRF does not exert its effect in the sensitization phase alone. OVA challenge is necessary for HRF to exert a local effect in the response phase as measured by increases in BAL IgE levels. In contrast, either OVA sensitization or OVA challenge will suffice for HRF to have an effect on serum IgE levels. In general, HRF augments the inflammatory response to allergens. This has been seen *in vitro* in human studies over the past several years and is now seen in the mouse model of allergic inflammation. Future studies with this transgenic mouse model will elucidate the specific mechanisms of HRF's actions on the inflammatory process with particular emphasis on the mouse basophil.
